# Gastrointestinal (GI) Tract Microbiome-Derived Neurotoxins—Potent Neuro-Inflammatory Signals From the GI Tract via the Systemic Circulation Into the Brain

**DOI:** 10.3389/fcimb.2020.00022

**Published:** 2020-02-12

**Authors:** Walter J. Lukiw

**Affiliations:** ^1^LSU Neuroscience Center, Louisiana State University Health Sciences Center, New Orleans, LA, United States; ^2^Department of Ophthalmology, Louisiana State University Health Sciences Center, New Orleans, LA, United States; ^3^Department of Neurology, Louisiana State University Health Sciences Center, New Orleans, LA, United States

**Keywords:** Alzheimer's disease (AD), *Bacteroides fragilis* and BFT (fragilysin), dysbiosis, lipopolysaccharide (LPS), microbiome and microbial genetics, neurofilament light (NF-L), neuroinflammation, synapsin-2 (SYN2)

## Abstract

The microbiome of the human gastrointestinal (GI)-tract is a rich and dynamic source of microorganisms that together possess a staggering complexity and diversity. Collectively these microbes are capable of secreting what are amongst the most neurotoxic and pro-inflammatory biopolymers known. These include lipopolysaccharide (LPS), enterotoxins, microbial-derived amyloids and small non-coding RNA (sncRNA). One of the major microbial species in the human GI-tract microbiome, about ~100-fold more abundant than *Escherichia coli*, is *Bacteroides fragilis*, an anaerobic, rod-shaped Gram-negative bacterium that secretes: (i) a particularly potent, pro-inflammatory LPS glycolipid subtype (BF-LPS); and (ii) a hydrolytic, extracellular zinc metalloproteinase known as *B. fragilis* toxin (BFT) or *fragilysin*. Ongoing studies support multiple observations that BF-LPS and BFT (*fragilysin)* disrupt paracellular barriers by cleavage of intercellular proteins, such as E-cadherin, between epithelial cells, resulting in ‘*leaky*’ barriers. These defective barriers, which also become more penetrable with age, in turn permit entry of microbiome-derived neurotoxic biopolymers into the systemic circulation from which they can next transit the blood-brain barrier (BBB) and gain access into the brain. This short communication will highlight some recent advances in this extraordinary research area that links the pro-inflammatory exudates of the GI-tract microbiome with innate-immune disturbances and inflammatory signaling within the human central nervous system (CNS) with reference to Alzheimer's disease (AD) wherever possible.

## Overview–The Human Gastrointestinal (GI) Tract Microbiome—*Bacteroidetes*

The gastrointestinal (GI)-tract microbiome, the largest reservoir of microbes in the human body, containing about 10^14^ microbial cells, is a complex, dynamic and abundant source of bacteria, methanogenic archaea, fungi, microbial eukaryotes, protozoa, viruses, and other microorganisms. Together the GI-tract microbiome possesses a remarkable microbiological diversity and staggering genetic complexity of at least 1000 major bacterial species. In the most recent estimate analyses of the GI-tract metagenomes of ~2100 donors well over 22.3 million non-redundant prokaryotic genes were detected, and at least half of all the genes identified were unique to an individual (Tierney et al., 2019). When compared to the established human genome content of 26.6 thousand protein-encoding transcripts of the human genome sequencing project obtained about ~18 years ago (Fields et al., 1994; Venter et al., 2001; Hicks et al., 2019) the number of microbial genes in the human GI-tract microbiome alone outnumbers human genes by about 837 to 1 (Tierney et al., 2019). Another interesting fact is that of the 52 major divisions of bacteria identified to date, only 2 phyla are known to predominate in the human GI-tract microbiome—the Gram-negative *Bacteroidetes* (representing about ~20–30% of all GI-tract resident bacteria) and the Gram-positive *Firmicutes* (representing ~70–80% of the total) with relatively minor contributions by *Actinobacteria* (~3%*), Proteobacteria* (~1%)*, Fusobacteria* (~0.1%) and *Verrucomicrobia* (0.1%). Collectively these microorganisms represent: (i) “the microbial core” of the human GI-tract microbiome (Sarkar and Banerjee, 2019; Ticinesi et al., 2019); (ii) an extremely active, dynamic, and changing ecosystem dependent on the host's age, diet, environment, ethnicity, and health and/or disease status (Sender et al., 2016; Zhao and Lukiw, 2018a,b; Rinninella et al., 2019); (iii) a rich source of commensal bacteria usually beneficial to human health because of their abilities to metabolize and/or biosynthesize complex sugars, polysaccharides, and dietary fiber into volatile short chain fatty acids (SCFAs; including acetate, propionate, butyrate, valerate and lactate and other nutrients). SCFAs (i) normally function in the development, maintenance, and homeostasis of the host immune, neuro-endrocrine and digestive systems; and (ii) play important regulatory roles in glucose homeostasis, lipid metabolism and anti-inflammatory signaling in endothelial cells of the lining of the GI-tract, sometimes known as the intestinal endothelium. Interestingly, there is recent evidence that SCFAs can signal through G-protein coupled receptors (GPCRs) at the cell surface, including GPCR41, GPCR43, and GPCR109a and these activate signaling cascades that control multiple immune functions. Recent transgenic mouse studies support a key role of these GPCRs in the regulation of intestinal inflammation (Sears, 2009; Fathi and Wu, 2016; Lukiw, 2016a,b; Castillo-Álvarez and Marzo-Sola, 2019; Fox et al., 2019; Parada Venegas et al., 2019).

Over 99% of the microbes in the human GI-tract are facultative and obligate anaerobic bacteria; the most abundant Gram-negative bacterial Phylum in the human GI-tract microbiome are the *Bacteroidetes*, with a major Genus-species being represented by the obligate Gram-negative anaerobe *Bacteroides fragilis*. In some intestinal tract regions *B. fragilis:* (i) are present at about ~100-fold the abundance of the *Proteobacteria Escherichia coli*; (ii) colonize the human GI-tract at densities up to 8 × 10^10^ CFU per cm^3^, the highest density of any microbial colonization known in nature (Sears, 2009; Fathi and Wu, 2016; Rios-Covian et al., 2017; Patrick et al., 2019; Rinninella et al., 2019); and (iii) reside and proliferate exclusively in the GI-tract of mammals, suggesting a strong adaptation to the pH, biophysical and microbial composition of the gut environment (Bhattacharjee and Lukiw, 2013; Wexler and Goodman, 2017; Poeker et al., 2018; Castillo-Álvarez and Marzo-Sola, 2019).

## Gi-Tract Exudates—BF-LPS and Fragilysin

In the human GI-tract there are 2 predominant strains of *Bacteroides fragilis* (*B. fragilis*) distinguished in part by their biosynthetic capabilities to synthesize and secrete a zinc-dependent metalloprotease toxin known as *B. fragilis* toxin (BFT) or *fragilysin*. Strains of *Bacteroides* that do not secrete BFT are called non-toxigenic *B. fragilis* while those that do secrete are called enterotoxigenic *B. fragilis* (ETBF; Allen et al., 2019). Relatively recently it has been established that enterotoxigenic strains of *B. fragilis* (ETBF) can rapidly proliferate in the mammalian GI-tract both in the absence of adequate dietary fiber and in the presence of high-fat cholesterol diets (Heinritz et al., 2016; Wexler and Goodman, 2017; Poeker et al., 2018; Zhao and Lukiw, 2018a,b). This proliferation enhances the intestinal abundance of *B. fragilis* and hence the potential of this Gram negative obligate anaerobe to secrete its formidable array of neurotoxic exudates. These primarily include: (i) the lipoglycan lipopolysaccharide (LPS), a particularly potent, pro-inflammatory LPS glycolipid subtype (BF-LPS); and (ii) the hydrolytic, extracellular zinc metalloproteinase known as ETBF-secreted *Bacteroides fragilis* toxin (BFT), also known as *fragilysin*. Recent characterization of BF-LPS and *fragilysin* have shown them to be amongst the most pro-inflammatory lipoglycans and enterotoxins known (Vines et al., 2000; Sears, 2009; Lukiw, 2016a,b; Zhao and Lukiw, 2018a,b; Batista et al., 2019; Sheppard et al., 2019). Both BF-LPS and *fragilysin* can leak through the normally protective mucosal barriers of the GI-tract intestinal endothelium to induce substantial inflammatory pathology both systemically and after BBB transit into vulnerable CNS compartments, including the neocortical parenchyma of the brain (Fathi and Wu, 2016; Lukiw, 2016a,b; Zhao and Lukiw, 2018a,b; Barton et al., 2019; Batista et al., 2019; Fox et al., 2019; Sheppard et al., 2019; Zhao et al., 2019). Indeed, while *Bacteroides fragilis* is an anaerobic, Gram-negative, rod-shaped bacillus, and part of the normal microbiota of the human colon and is generally commensal, this microbe can cause a “smoldering” systemic infection if displaced into the bloodstream or surrounding tissue following disease, trauma or surgery (Hill et al., 2014a,b; Montagne et al., 2017; Tulkens et al., 2018; Erdo and Krajcsi, 2019; Fox et al., 2019; Patrick et al., 2019; Sarkar and Banerjee, 2019; Sweeney et al., 2019). When the highly toxic exudates of enterotoxigenic strains of *B. fragilis* escape the microbial-dense environment of the human GI-tract they can produce substantial systemic inflammatory pathology with significant mortality and morbidity. *B. fragilis* proliferation is associated with, and causative for, bacteremia, brain and intra-abdominal abscess, cellulitis, colitis, diabetic ulcer, diarrhea, necrotizing fasciitis, sepsis, peritonitis, septicemia, association with and the development of multiple pro-inflammatory bowel cancers, systemic infection and systemic inflammation, the development of neurological diseases involving inflammatory neurodegeneration, and those neurological disorders that display a significantly elevated incidence of atypical developmental programming against a background of aging (Leshchyns'ka and Sytnyk, 2016; Agrawal et al., 2017; Shivaji, 2017; Zhao et al., 2019). Very recently LPS-induced systemic inflammation has been associated with synaptic loss and cognitive decline in multiple human neurological disorders and in animal models, and a role for LPS-mediated microglial release of pro-inflammatory cytokines (such as IL-1β) based on both *in vivo* and primary culture studies *in vitro* (Sheppard et al., 2019; Zhao et al., 2019).

## Gastrointestinal (GI)-Tract and Blood Brain Barrier (BBB) Dysfunction

**Figure 1:**
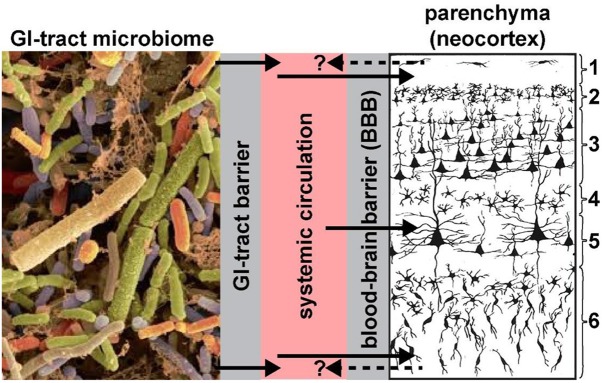
Highly schematicized depiction of the potential transfer of GI-tract microbiome-derived pro-inflammatory neurotoxins across the GI-tract barrier into the systemic circulation, followed by translocation across the BBB into the brain parenchyma (see Hill et al., 2014a,b; Hill and Lukiw, 2015; Zhao and Lukiw, 2018a; neocortical region shown; solid black arrows). Neurotoxins identified to date include *Bacteroides fragilis* lipopolysaccharide (BF-LPS) and enterotoxins such as the *Bacteroides fragilis*-derived toxin (BFT) also known as *fragilysin*. The contribution of other pro-inflammatory neurotoxins such as GI-tract-derived amyloids and bacterial sncRNAs are not well-understood and currently very little is known concerning their neurotoxicity and CNS-effects. BF-LPS, BFT (*fragilysin*), age, dietary toxins, traumatic brain injury (TBI) and vascular disease are known to effectively disrupt endothelial cell-based biophysical barriers in part through the cleavage, disruption and/or degeneration of cell-cell adhesion proteins (Wu et al., 1998; Clement et al., 2016; Zhao and Lukiw, 2018a,b; Sweeney et al., 2019). We speculate that all of these neurotoxins together have potential to constitute a highly neurotoxic pro-inflammatory GI-tract microbiome-derived cocktail greatly detrimental to the cytoarchitecture and signaling functions of neuronal and glial cells. Gram-negative bacterial-derived LPS and related neurotoxins have been recently observed within the systemic circulation and brain parenchyma (Zhan et al., 2016, 2018; Zhao et al., 2017a,c, 2019; Zhao and Lukiw, 2018a), within and around neurons, and in the later stages of AD completely encapsulating neuronal neocortical nuclei (Hill et al., 2014b,a; Hill and Lukiw, 2015; Zhao and Lukiw, 2018a,b; unpublished observations); this later action appears to impair the exit of neuron-specific transcripts such as the neurofilament-light (NF-L) chain and synapsin-2 (SYN2) messenger RNA (mRNA) from the neuronal nuclei; both NF-L and SYN2 mRNA abundance and expression are down-regulated in LPS-treated human neuronal-glial (HNG) cells in primary culture and in AD brain (Lukiw et al., 2018; Zhao et al., 2019). Whether neurotoxins from the brain parenchyma of the neocortex can cross the BBB back into the systemic circulation (dashed black arrows with question mark) is currently not well-understood; if so, these species may be useful serum biomarkers for both the diagnosis and prognosis of AD and other types of inflammatory neurodegeneration; left panel = GI-tract microbiome magnification x ~3000 (source: http://www.Injnbio.com; http://www.lnjnbio.com/nd.jsp?id=20; permission to reproduce granted; last accessed 26 November 2019); right panel = 6-layered structure of the human association neocortex; layers 3 and 5 are the pyramidal cell layers targeted by the AD process; other brain regions may also be affected; magnification x ~20 (source: adapted and redrawn from Martinez-Conde, 2018; last accessed 26 November 2019).

Two anatomical gateways, including the gastrointestinal mucosa that includes the “GI-tract barrier” and the “blood-brain barrier (BBB),” each formed essentially by vascular epithelial and/or endothelial cells and epithelial-endothelial-derived basement membranes provide both a biophysical interface and a biological compartmentalization of the GI-tract microbiome, the systemic circulation, the brain parenchyma and distinct anatomical regions of the brain such as the neocortex (Figure 1). These barriers are a requisite for the essential maintenance of homeostasis and the physiological environment of each compartment; microorganisms of the GI-tract microbiome and their neurotoxins that are able to transit the single layer of epithelial cells have virtually unimpeded access into the systemic circulation (Varatharaj and Galea, 2017; Logsdon et al., 2018; Sweeney et al., 2019; Tulkens et al., 2018). Probably the most important structural components of these barriers are the multiple tight junctions between adjacent cells of vascular capillaries (Sweeney et al., 2018, 2019; Tulkens et al., 2018). For example, the blood-brain barrier of the CNS can selectively regulate its intracellular compartments and thereby isolate itself from rapid biochemical or biophysical changes that may occur in the systemic circulation.

The surface area of the human GI-tract barrier and the BBB is remarkably large; for example although the interior of the small intestine is only about ~3.5 cm in diameter and ~7 m in length (due in large part to a concentrically folded mucosa) it has a total absorptive surface area of ~32 m^2^ (Helander and Fändriks, 2014; Sweeney et al., 2019). Similarly the surface area of the 600 km of the human brain's 5 um diameter microvessels, representing the majority of the BBB, corresponds to a total surface area of ~25 m^2^ (Wong et al., 2013). Hence the maintenance of these large and formidable biophysical barriers is a very active and energy-intense biological process (Wong et al., 2013; Varatharaj and Galea, 2017; Barton et al., 2019; Erdo and Krajcsi, 2019; Sweeney et al., 2019). BF-LPS, BFT (*fragilysin*) and other GI-tract microbiome-derived exudates are remarkable in their capabilities: (i) of breaking down the intercellular junctions of these barriers via their disruptive actions on cadherins and other cell-cell adhesion molecules (Wu et al., 1998; Wong et al., 2013; Sweeney et al., 2018); (ii) of altering BBB integrity and permeability (Varatharaj and Galea, 2017; Tulkens et al., 2018; Sweeney et al., 2019); (iii) of changing BBB transport rates (Wong et al., 2013; Logsdon et al., 2018); (iv) of modulating neuroimmune signaling or the transport of immune-regulatory molecules (Erdo and Krajcsi, 2019); (v) of trafficking dietary neurotoxins and pathogens into the brain (Sweeney et al., 2019; Wong et al., 2013); and/or (vi) of inducing the release of inflammatory and neuro-immune substances from the barrier cells (Hill et al., 2014a,b; Köhler et al., 2016; Lukiw, 2016a,b; Montagne et al., 2017; Varatharaj and Galea, 2017; Tulkens et al., 2018; Sweeney et al., 2018, 2019; Erdo and Krajcsi, 2019; Fox et al., 2019; Patrick et al., 2019; Sarkar and Banerjee, 2019).

Indeed, the most recent research evidence continues to strengthen the idea that one major, and virtually unlimited, source of pro-inflammatory neurotoxic signals in inflammatory neurodegeneration such as those typified by the AD process may originate from internally derived noxious exudates such as those supplied via the diet and metabolized by the GI-tract microbiome (Sweeney et al., 2018, 2019; Erdo and Krajcsi, 2019). Because of aging, traumatic brain injury (TBI), cerebrovascular deficits (some of which may be genetic), neurovascular pathology or neuroinflammatory brain degeneration, neurotoxic molecules can “leak” into the systemic circulation, prompting some investigators to propose that progressive neurodegenerative diseases such as AD supplied via the reflect a malfunction of key biophysical barriers including those of the GI-tract and BBB, and that AD is in fact a “*defective barrier”* disease (Bhattacharjee and Lukiw, 2013; Montagne et al., 2017; Sweeney et al., 2018, 2019; Erdo and Krajcsi, 2019). The abundance of blood-borne bacterial components including LPS, for example, represents a variable component of the human blood serum that can elicit variable systemic pro-inflammatory and innate-immune-modulatory responses in the host, resulting in systemic-immune activation by pathogen-associated molecular patterns (PAMPs), a process sometimes referred to as “*microbial translocation*” (Zhao et al., 2017a,b; Tulkens et al., 2018; Di Lorenzo et al., 2019; Logsdon et al., 2018; Patrick et al., 2019). Multiple recent reports further suggest that GI-tract dysbiosis and “leaky gut syndrome” constitute a vastly under-appreciated, under-studied and critical pathophysiological passageway for transport of GI-tract microbiome-derived neurotoxins across GI-tract and blood–brain biological barriers resulting in an age-related progression from systemic inflammation to neurovascular disease to CNS inflammation and degeneration that progressively contribute to critical aspects of neuropathology associated with age-related neurodegenerative disorders. These include neuropathological disorders such as AD, anxiety, autism spectrum disorder (ASD), depression, epilepsy, multiple sclerosis, Parkinson's disease (PD), prion disease, systemic inflammatory response syndrome, and other incapacitating and/or ultimately lethal neurological diseases of the human CNS (Hill et al., 2014a,b; Köhler et al., 2016; Li and Yu, 2017; Varatharaj and Galea, 2017; Zhao et al., 2017a,b, 2019; Griffiths and Mazmanian, 2018; Di Lorenzo et al., 2019; Fox et al., 2019; Patrick et al., 2019; Sarkar and Banerjee, 2019).

## BF-LPS and the Induction of the Pro-Inflammatory Transcription Factor NF-κb and microRNA-146a

The extruded lipopolysaccharide shed from the human GI-tract microbiome-abundant *Bacteroides fragilis* (BF-LPS): (i) is one of the most pro-inflammatory and neurotoxic lipoglycans known (Sears, 2009; Fathi and Wu, 2016; Lukiw, 2016a,b; Allen et al., 2019); (ii) is linked to synaptic loss and cognitive decline in human patients and in animal models (Sheppard et al., 2019); and (iii) is recognized by the Toll receptors TLR2, TLR4, and/or CD14 microglial cell receptors, as are the pro-inflammatory and hydrophobic 42 amino acid amyloid-beta (Aβ42) peptides whose accumulation are a characteristic feature of AD brain (Sears, 2009; Zhao et al., 2015; Zhao and Lukiw, 2015; Lukiw, 2016a,b; Batista et al., 2019; Sheppard et al., 2019). Additional LPS-mediated pro-inflammatory actions, pathogenic mechanisms and neurodegeneration-promoting activities remain incompletely understood but remarkable progress is being made both in transgenic animal models and in human patient studies (Zhan et al., 2018; Zhao and Lukiw, 2018a,b; Barton et al., 2019; Sarkar and Banerjee, 2019; Sheppard et al., 2019; Wu et al., 2007). For example, LPS-induced synaptic loss and the impairment of cognition appear to be in part the result of a modified microglial activation, reactive oxidative species (ROS) or cytokine generation and oxidative stress damage, disruption of the intercellular adhesion proteins associated with the GI-tract or blood-brain barriers, the ROS mediated oxidation, atrophy, destruction and loss of synapse-related proteins, elevations in neuroinflammatory signaling or any combination of these events (Barton et al., 2019; Batista et al., 2019; Sheppard et al., 2019; Wu et al., 2007). One recently described BF-LPS mediated pathogenic and AD-relevant pathway is the robust activation of NF-κB (p50/p65) in human brain cells in primary culture and induction of a pro-inflammatory signaling pathway involving an NF-kB-regulated microRNA-146a, and the subsequent chronic and pathogenic over-stimulation of innate-immune and neuro-inflammatory pathways (Zhao and Lukiw, 2018b). These include deficits in the innate-immune system modulator complement factor H (CFH), decreases in the expression of the essential presynaptic neuronal phosphoprotein synapsin-2 (SYN2) and down-regulation of the neuron-specific neurofilament light chain (NF-L) cytoskeletal protein (Lukiw et al., 2018; Zhan et al., 2018; Zhao et al., 2019). These pathological signaling pathways appear to strongly contribute to synaptic disorganization and decline, neuronal atrophy and inflammation-mediated amyloidogenic neuropathology which are all characteristic attributes of the AD-affected brain.

## Unanswered Questions

The neurobiological signaling connections between the GI-tract microbiome and CNS disease remain incompletely understood. Eighteen years after the elucidation and characterization of the genes expressed in the human genome (Venter et al., 2001), the staggering genetic complexity of the human GI-tract and oral microbiomes have been analyzed with remarkable and unexpected results (Tierney et al., 2019). It is truly extraordinary that the potential contribution to human health and disease by the GI-tract microbiome with a total mass, complexity and diversity, and number of genes exceeding that of the liver could have been almost completely overlooked as recent as just ~10 years ago.

Several fundamental questions remain concerning nature of the microorganisms of the GI-tract microbiome, their compartmentalization within the GI-tract and their potential effects on the neuropathology, neurobiology, and the pathogenetics of inflammatory neurodegeneration and neuropsychiatric disease. It will be further interesting to discover: **(**i) the evolutionary history of the GI-tract microbiome and for example, why just 2 of 52 bacterial phyla were selected and evolved to be both dominant and symbiotic within the entire human metagenome; (ii) what patterns of microbial abundance, speciation, complexity, stoichiometry, dysbiosis and GI-tract-derived mixtures of neurotoxins are the most effective in promoting pathogenic inflammatory neuro-degeneration; (iii) if the incidence of blood-borne GI-tract-derived toxic elements in the systemic inflammation could be used as a pathological biomarker or be of prognostic value for AD and other progressive, age-related, neurodegenerative diseases; (iv) what would be the contribution of combinations of the microbial constituents including archaebacteria, fungi, protozoa, viruses, and other GI-tract resident microbes of the GI-tract microbiome to enhance neurological health; (v) the intriguing possibility that the composition of the GI-tract microbiome could be altered through diet, probiotics and/or prebiotics to optimize human neurological health; (vi) if the penetration of epithelial barriers by bacterial products occurs in the oral cavity in periodontal disease with similar systemic effects; (vii) the mechanism of the duality of GI-tract abundant Gram-negative anaerobic bacteria such as *B. fragilis* in behaving in both pro- and anti-inflammatory capacities, the role of capsular polysaccharides and IL-10 secreting B and T cells in this transition, and how the role of *B. fragilis* can switch from an abundant beneficial microbe and commensal microorganism to a highly neurotoxic one (Ramakrishna et al., 2019); and (viii) perhaps most importantly, if medical researchers along with neurologists and dieticians could devise a strategy, perhaps through “*personalized medicine*,” that promotes the lowering of noxious GI-tract microbes and their secretions that would optimize life-long GI-tract microbiome function and CNS health. This approach might minimize the risk of developing AD and other highly incapacitating human diseases as we age. Furthering our molecular-genetic and mechanistic understanding of how different secreted components of the GI-tract microbiome negatively affect the CNS may uncover potential and novel strategic approaches for the GI-tract microbiome-based modulation of neurological function, and the more effective clinical management of terminal, age-related neurological disorders.

## Concluding Remarks

The appreciation of a potential contribution from the GI-tract microbiome to human neurological health and devastating behavioral, amnestic and cognitive disorders such as AD is a relatively recent one (Bhattacharjee and Lukiw, 2013), and gathering recent evidence continues to strengthen this association (Johnson and Foster, 2018; Patrick et al., 2019; Sarkar and Banerjee, 2019; Sheppard et al., 2019; Strandwitz et al., 2019; Sweeney et al., 2019; Ticinesi et al., 2019; Tierney et al., 2019; Zhao et al., 2019). Dietary manipulations of the GI-tract microbiome including diets enriched in biologically soluble and insoluble fiber, that seem to neutralize the potentially neurotoxic secretions from Gram-negative bacilli such as *Bacteroides fragilis* might provide a life-long resolution to defer the development of human neuro-inflammatory degenerative disease (Heinritz et al., 2016; Chen et al., 2017; Poeker et al., 2018). It is becoming increasingly established that the contribution of the GI-tract microbiome and GI-tract microbiome-derived neurotoxins to pathogenic signaling associated with inflammatory neurodegeneration is: (i) age-related and progressive; (ii) contains multiple neurotoxic components with capability to breach biophysical barriers; (iii) constitute a virtually unlimited supply of BF-LPS, BFT (*fragilysin)* and other neurotoxins; and (iv) that the GI-tract microbiome comprises a “staggering” microbial genetic complexity, and the recent finding that at least half of all the genes identified are unique to each individual further underscores the interesting parallel in the heterogeneity between GI-tract microbiome composition and AD risk, onset and development (Patrick et al., 2019; Strandwitz et al., 2019; Ticinesi et al., 2019; Tierney et al., 2019). Of further recent interest is the potential involvement of the GI-tract microbiota-brain axis with the mental status of the host in that certain “*psychotropic bacteria”* and their secreted array of “*psychobiotics*” appear to influence the mental health of the host (Beck et al., 2019; Cheng et al., 2019; Kelly et al., 2019). Given that AD was originally referred to as a progressive and dementing “*senile psychosis*,” efficacious manipulation of the GI-tract microbiome might not only attenuate inflammatory neurodegeneration, synaptic disorganization and cognitive decline but also optimize healthy neuroimmune, neuroendocrine, humoral and brain signaling pathways that also promote well-being, anti-depressive and anxiolytic behaviors in patients affected by the AD process.

## Data Availability Statement

The datasets generated for this study are available on request to the corresponding author.

## Ethics Statement

The studies involving human participants were reviewed and approved by Louisiana State University Institutional Review Board—only post-mortem human tissues were used in these studies. The ethics committee waived the requirement of written informed consent for participation.

## Author Contributions

WL distilled the results from all laboratory experiments at the LSU laboratories and performed literature searches of recent peer-reviewed publications in this research area, compiled all data, and wrote this manuscript.

### Conflict of Interest

The author declares that the research was conducted in the absence of any commercial or financial relationships that could be construed as a potential conflict of interest.
